# Unveiling the therapeutic potential of berberine: its therapeutic role and molecular mechanisms in kidney diseases

**DOI:** 10.3389/fphar.2025.1549462

**Published:** 2025-02-21

**Authors:** Zhongyu Fan, Xuejiao Wei, Xiaoyu Zhu, Kun Yang, Ling Tian, Xiaoyan Wang, Yujun Du, Liming Yang

**Affiliations:** Department of Nephrology, The First Hospital of Jilin University, Changchun, China

**Keywords:** berberine, renal diseases, inflammation, oxidative stress, apoptosis

## Abstract

Berberine (BBR) is a pentacyclic benzylisoquinoline alkaloid widely distributed across various medicinal plants. Recent studies have demonstrated that berberine possesses a broad spectrum of pharmacological activities, including not only antioxidant properties but also the ability to lower blood glucose, modulate lipid profiles, and mitigate inflammation. These findings suggest that berberine holds significant potential as a therapeutic agent for renal diseases, highlighting its substantial research value. Moreover, when administered orally, berberine has been shown to exhibit a wide therapeutic safety margin. Several studies have identified berberine’s renoprotective effects across a range of kidney disorders, including diabetic nephropathy, renal fibrosis, renal aging, kidney toxicity induced by chemotherapy and antibiotics. These properties underscore berberine’s evolving therapeutic potential for both acute kidney injury (AKI) and chronic kidney disease (CKD). In summary, the research discussed in this article provides a comprehensive overview of the renoprotective effects of BBR and elucidates the molecular mechanisms underlying its therapeutic potential in the treatment of various renal disease. Furthermore, the article underscores the significance of berberine as a promising therapeutic agent for the treatment of kidney disorders.

## Graphical Abstract

**Figure:**
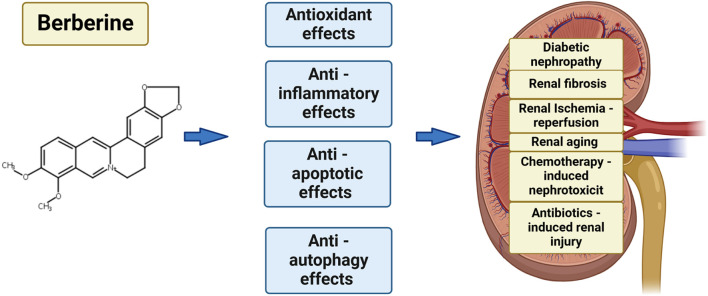

## 1 Introduction

Renal damage is generally categorized into two main forms: acute kidney injury (AKI) and chronic kidney disease (CKD). The etiopathogenesis of intrinsic AKI may be attributed to various factors, including renovascular causes resulting from conditions such as vasculitis or vascular stenosis, glomerular involvement typically linked to immune complex-mediated diseases like systemic lupus erythematosus, and interstitial damage induced by inflammation, ischemia or nephrotoxic agents such as chemotherapeutic agents, nonsteroidal anti-inflammatory drugs (NSAIDs), and aminoglycoside (Radi, 2018; Radi et al., 2018a; Radi et al., 2018b). CKD affects approximately 10%–13% of the global population and is distinguished by its insidious, progressive course, which frequently culminates in an increased susceptibility to cardiovascular complications. In its early stages, CKD typically remains asymptomatic, with patients only exhibiting symptoms related to renal failure in advanced stages (Jager and Fraser, 2017; Levey and Coresh, 2012). Despite the availability of advanced diagnostic and supportive interventions, AKI and CKD continue to exhibit high morbidity and mortality rates, primarily due to the limited efficacy of current therapeutic options. Consequently, there is a critical need to identify and develop more effective therapeutic strategies to both treat and mitigate the progression of renal diseases.

BBR is an alkaloid compound belonging to the isoquinoline class, commonly utilized in traditional Chinese and Ayurvedic medicine. It is derived from a range of plant species within the *Berberidaceae*, *Papaveraceae*, and *Ranunculaceaefamilies*. BBR has garnered significant attention owing to its broad range of pharmacological effects and diverse mechanisms of action. The earliest documented use of *Rhizoma coptidis*, a source of berberine, for medicinal purposes dates back to the year 200 A.D (Feng et al., 2019). BBR is a bioactive alkaloid with a millennia-long history of therapeutic applications in traditional medicine. It demonstrates a wide array of therapeutic properties, including antioxidative, anti-inflammatory, and anti-apoptotic activities (Lin and Zhang, 2018; Wang et al., 2017). Recent studies have increasingly highlighted berberine’s potential to confer protective effects against ischemia-reperfusion (I/R) injury in multiple organs, including the heart, brain, kidneys, intestines, liver,and testes (Kazaz et al., 2020; Gu et al., 2013). BBR has demonstrated nephroprotective effects across a diverse spectrum of renal disorders, including those associated with ischemia-reperfusion injury (Zhang et al., 2020), kidney fibrosis (Shao et al., 2021), medication or toxin induced injury (Ibrahim Fouad and Ahmed, 2021), kidney stones (Bashir and Gilani, 2011) and kidney aging (El-Horany et al., 2020). This review delves into the molecular pathways underlying the renoprotective properties of berberine in the context of various nephropathies.

## 2 Berberine

### 2.1 Chemical and physical properties of BBR

BBR is readily extracted from various traditional medicinal plants, where it is broadly dispersed across the roots, stems, and bark of species with notable pharmacological value. These include *Berberis vulgaris, Coptis chinensis and Berberis aristata*. The genus Berberis represents the most extensively distributed natural reservoir of BBR, with its concentration in medicinal plants ranging significantly from 0.05 mg/g to 96.10 mg/g. Among documented species, *C. chinensis* exhibits the highest BBR content, followed by *Berberis asiatica* and *Coptis teeta* (Lu et al., 2018). Barberry and other berberine-rich plants have been integral to nearly all traditional medical systems, with their therapeutic use tracing back over 3,000 years in Ayurvedic, Iranian, Chinese and Egyptianand medicine practices (Sarraf et al., 2019).

BBR is a chemically stable quaternary ammonium isoquinoline alkaloid, characterized by the molecular formula C_20_H_18_NO_4_ and the molecular weight is 336.37 g/mol (Feng et al., 2019). Free BBR exhibits limited solubility in water, dissolves readily in hot ethanol, and shows minimal solubility in low-polarity organic solvents for instance chloroform and benzene. Its hydrochloride form has reduced water solubility but dissolves more efficiently in boiling water, while its phosphate derivatives and sulfate are comparatively more water-soluble (Feng et al., 2019).

Despite its extremely low concentration in the bloodstream (Hua et al., 2007), BBR exhibits a bioavailability of less than 1% (Kheir et al., 2010), the pharmacological efficacy of BBR is closely associated with its extensive distribution across tissues. Previous studies have demonstrated that BBR readily crosses the blood–brain barrier, exhibiting rapid accumulation in the hippocampus following intravenous administration, with subsequent slow clearance (Wang et al., 2005). Moreover, research has demonstrated that the levels of BBR and its pharmacologically active metabolites are significantly elevated in various organs compared to their levels in the bloodstream following oral administration. BBR exhibits rapid tissue distribution, with the highest accumulation observed in the liver, followed by the kidneys, lungs heart and pancreas, while minimal distribution occurs in adipose tissue, where levels remain relatively stable for up to 48 h (Tan et al., 2013).

Extensive scientific research indicates that berberine undergoes metabolic transformation primarily through demethylation, glucuronidation, and sulfonation (Ahmed et al., 2015). Notably, the pharmacological properties of its metabolites align closely with those of the parent compound (Wang et al., 2017). The primary active metabolites of BBR can be classified into four major types: berberrubine, thalifendine, demethyleneberberine, and jatrorrhizine (Liu et al., 2009). The oral bioavailability of berberrubine may surpass that of BBR. These findings suggest that investigating the metabolically active derivatives of berberine could offer a promising avenue for enhancing its therapeutic efficacy. Finally, with regard to excretion, BBR is predominantly eliminated via urine and bile in its metabolized forms. In addition, BBR exhibits a robust safety profile, with clinical trials reporting a paucity of adverse events (Cheng et al., 2022).

### 2.2 Pharmacological and therapeutic effects of BBR

**FIGURE 1:**
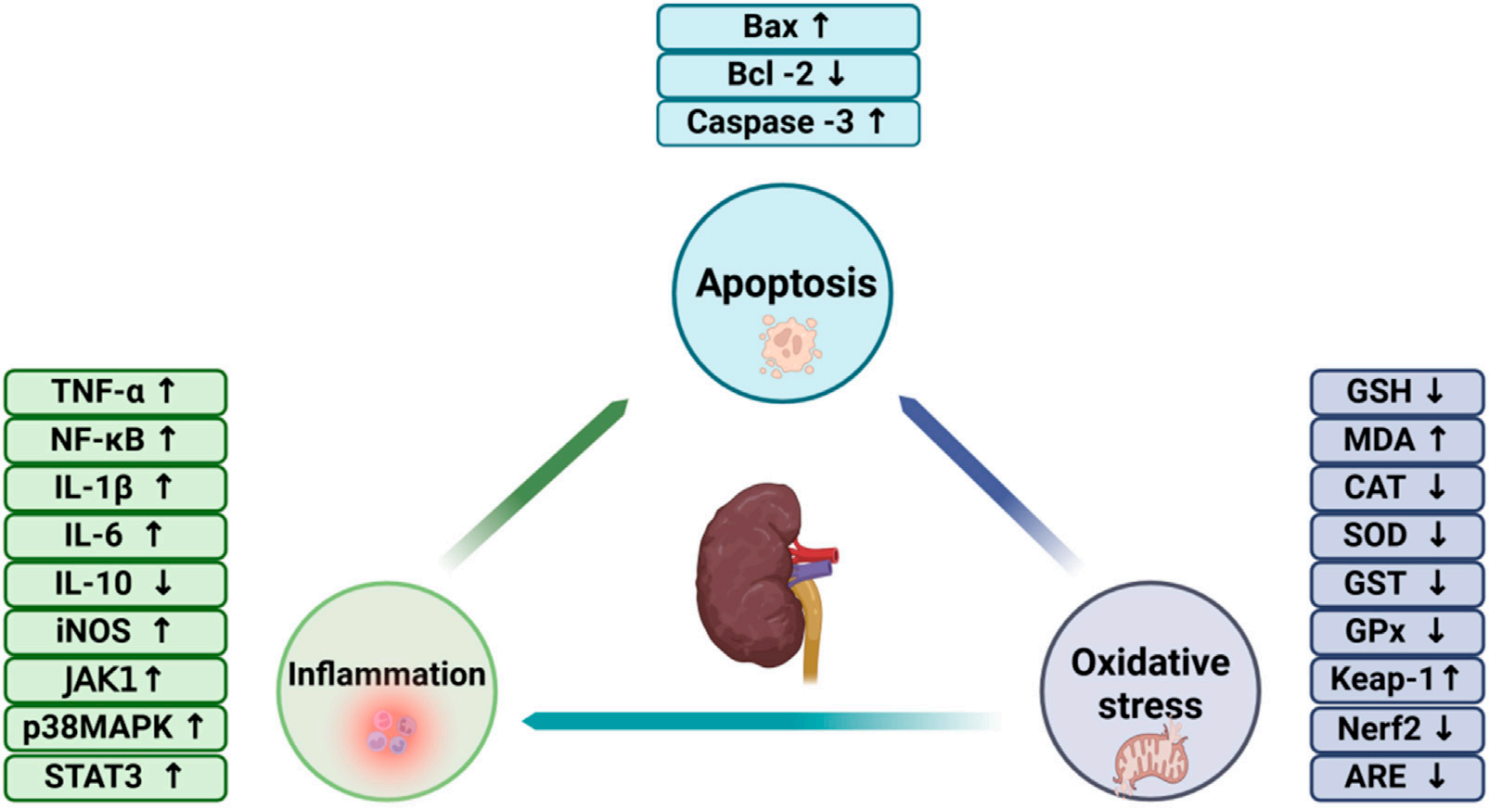
The involvement of oxidative stress, inflammation, and apoptosis in the pathogenesis of renal disorders.

For many years, comprehensive investigations have been undertaken to explore the biological activities of BBR, with clinical studies highlighting its diverse range of pharmacological properties. Numerous studies have emphasized the therapeutic potential of BBR across various domains, including its roles as an anticancer (Nishino et al., 1986), antihyperglycemic (Jiang et al., 2015), antioxidant (Shirwaikar et al., 2006), and anti-inflammatory (Kuo et al., 2004). Additionally, numerous studies have demonstrated that BBR possessescardioprotective, hepatoprotective and neuroprotective properties. Notably, BBR has shown nephroprotective efficacy in various models of AKI and CKD, underscoring its potential as a therapeutic agent for renal disorders (Figure 1).

#### 2.2.1 Oxidative stress and renal diseases

At physiological concentrations, reactive oxygen species (ROS) function as key signaling mediators, orchestrating a variety of critical cellular processes (Finkel, 2011). However, excessive ROS production perturbs redox balance, resulting in oxidative damage to key biomolecules, including DNA, lipids and proteins (Liguori et al., 2018). It is broadly estimated that individual human cells experience approximately 10^5^ oxidative assaults daily from ROS, including hydroxyl radicals. Hydroxyl radicals (Valko et al., 2004), capable of interacting with all components of DNA are the principal agents driving DNA damage (Lombardi et al., 1998). Such damage results in genetic alterations, including chromosomal rearrangements and mutations, which can ultimately drive cellular senescence. Renal tubular epithelial cells are closely linked to the progression of renal senescence (Guyton and Kensler, 1993). Elevated levels of tubular epithelial cell senescence have been observed in animal models of AKI induced by ischemia-reperfusion injury (IRI) and CKD caused by unilateral ureteral obstruction (UUO), as well as in patients with CKD (Kim et al., 2021). Senescent cells secrete a range of cytokines, including transforming growth factor-β (TGF-β), interleukin-1β (IL-1β), interleukin-6 (IL-6) and interleukin-8 (IL-8), collectively termed the senescence-associated secretory phenotype (SASP), which exerts profound effects on the function of adjacent cells and tissues (Takahashi et al., 2018; Freund et al., 2010). The SASP drives inflammatory and pro-fibrotic responses, stimulating the activation of renal interstitial fibroblasts. This activation leads to excessive matrix protein synthesis, disrupting the normal architecture of renal tissues and facilitating fibrotic scar formation (Xu et al., 2020).

#### 2.2.2 Inflammation and renal diseases

Inflammation, a central pathological mechanism underlying both AKI and CKD, is characterized by complex interactions among dendritic cells, macrophages, and circulating leukocytes. This process is closely linked to the activation of nuclear factor-κB (NF-κB) signaling and NOD-like receptor thermal protein domain associated protein 3 (NLRP3) inflammasomes, along with the release of pro-inflammatory cytokines, which can result in irreversible tissue damage (Andrade-Oliveira et al., 2019). What’s more, both *in vivo* and *in vitro* investigations have elucidated that NF-κB activation in resident glomerular cells plays a pivotal role in driving the progression of renal damage. Numerous stimuli trigger the activation of the canonical NF-κB pathway, which modulates the expression of various cytokines. Members of the TNF superfamily and angiotensin II (Ang II) are prominent activators of NF-κB in renal pathologies (Sanz et al., 2010). In the streptozotocin (STZ)-induced rat model of diabetic nephropathy, Zhu et al. reported that berberine alleviated renal injury by attenuating Toll-like receptor 4 (TLR4)-dependent NF-κB-mediated inflammation (Zhu et al., 2018). The chemokines and pro-inflammatory cytokines produced by tubular epithelial cells serve as key components of the stress response, as well as mechanisms involved in tissue repair and restoration. Nevertheless, persistent renal inflammation can lead to the progression of AKI and CKD. In systemic conditions like sepsis, tubular epithelial cells play a pivotal role in the overproduction and release of inflammatory mediators, which contribute to damage in both the kidneys and remote organs.

#### 2.2.3 Apoptosis and renal disease

Apoptosis, evident through podocyte depletion and tubular cell attrition, exacerbates the decline in renal epithelial cells, a hallmark of both AKI and CKD (Hughes et al., 2004; Lorz et al., 2006). Notably, the kidney appears to be particularly responsive to the protective effects of anti-apoptotic molecules (Lorz et al., 2006). The role of autophagy in AKI has been primarily explored in tubular epithelial cells and podocytes. Initial evidence, derived from a renal I/R injury rat model, revealed upregulation of autophagy-related proteins, including light chain 3 (LC3) and Beclin1, within proximal and distal epithelial cells. Moreover, it has been demonstrated that enhanced expression of B-cell lymphoma-XL (Bcl-XL) in the kidney effectively suppresses both autophagy activation and apoptotic processes (Sureshbabu et al., 2015).

Consistent with this finding, autophagosomes were detected in murine renal tubular cells following I/R injury. It has also been reported that autophagic flux is elevated during the reperfusion phase. Similarly, studies utilizing kidney tubule-specific autophagy-deficient mouse models have provided more conclusive evidence regarding this process. In podocytes, elevated basal autophagic activity is essential for maintaining normal cellular homeostasis (Mizushima et al., 2004). Conditional knockout of autophagy-related genes, including autophagy-related protein 5 (Atg5) or autophagy-related protein 7 (Atg7), in mice led to pronounced vacuolization in both podocytes and tubular cells, ultimately contributing to the development of focal segmental glomerulosclerosis and renal dysfunction (Kawakami et al., 2015).

## 3 The protective effects of BBR in kidney diseases

### 3.1 Diabetic nephropathy

Diabetic nephropathy (DN), a major microvascular complication of diabetes mellitus, is the foremost cause of end-stage renal disease (ESRD) worldwide (Yan et al., 2007). It is estimated that approximately one-third of individuals with diabetes, irrespective of whether they have type 1 or type 2 diabetes, are affected by this condition. In the United States, 20%–40% of individuals with diabetes develop DN, which is the leading cause of ESRD (Reutens and Atkins, 2011).

Hyperglycemia impairs renal capillary dilation, induces podocyte depletion, and triggers oxidative stress within the nephron’s tubular system. Additionally, as filtrate albumin levels rise, excessive tubular reabsorption ensues, leading to inflammatory and fibrotic responses that contribute to the progressive decline of renal function (Ibrahim and Hostetter, 1997). The anti-inflammatory and antifibrotic effects of BBR play a role in alleviating diabetic nephropathy.

Initially, the inhibition of the NF-κB signaling pathway is observed. In a STZ-induced DN rat model, BBR alleviated renal damage by reducing fasting blood glucose levels, kidney-to-body weight ratio, 24-h proteinuria, creatinine (Cr) and blood urea nitrogen (BUN) concentrations. BBR mitigated systemic and renal cortical inflammation in STZ-induced DN models and high glucose (HG)-stimulated podocytes by downregulating the TLR4/NF-κB signaling pathway (Zhu et al., 2018). One study demonstrated that, in the context of diabetes, BBR reduces fibronectin (FN) expression in mesangial cells by targeting the sphingosine 1-phosphate (S1P) receptor subtype 2, an effect potentially linked to its suppression of NF-κB activation (Huang et al., 2012). Additionally, recent research highlights BBR’s ability to mitigate type 2 diabetes by attenuating NF-κB-mediated renal inflammation and inhibiting the TGF/Smad3 signaling pathway (Sun et al., 2015).

Secondly, mesangial cell proliferation was effectively attenuated. Excessive proliferation of glomerular mesangial cells represents a key pathological hallmark of DN. Recent research demonstrated that Huang-Gui solid dispersion, an innovative berberine-based formulation, mitigates diabetic nephropathy by inhibiting mesangial matrix expansion in the kidney and enhancing autophagic activity, potentially through the activation of Adenosine 5′-monophosphate (AMP)-activated protein kinase (AMPK) phosphorylation (Zhang et al., 2020).

Thirdly, berberine exhibits potent anti-inflammatory properties. In the kidneys of DN hamster models, the NLRP3–Caspase-1–Gasdermin D (GSDMD) signaling pathway was found to be upregulated. BBR mitigates oxidative stress-induced damage by enhancing the antioxidative activity of nuclear factor erythroid 2-Related Factor 2 (Nrf2), which subsequently modulates the NLRP3–Caspase-1–GSDMD axis. This regulation inhibits pyroptosis and counters inflammation-driven renal injury in DN (Ding et al., 2021). Berberine suppresses HG-induced epithelial-to-mesenchymal transition (EMT) and renal interstitial fibrosis by inhibiting the activation of the NLRP3 inflammasome. These findings suggest the potential of berberine as a novel therapeutic agent for managing tubulointerstitial fibrosis associated with diabetic nephropathy (Ma et al., 2022).

Fourthly, BBR exhibits anti-fibrotic properties. Proliferation of glomerular mesangial cells represents a key pathological feature in DN. In alignment with this, BBR mitigates renal tubular EMT and renal interstitial fibrosis by modulating the Notch/snail signaling pathway (Yang et al., 2017).

Despite these promising effects in DN, the underlying molecular mechanisms and specific pharmacological targets remain poorly elucidated. Consequently, future research should focus on identifying precise therapeutic targets and characterizing the alterations in downstream signaling pathways, enabling berberine to exert a pivotal role in the treatment of DN by targeting these pathways.

### 3.2 Renal fibrosis

**FIGURE 2:**
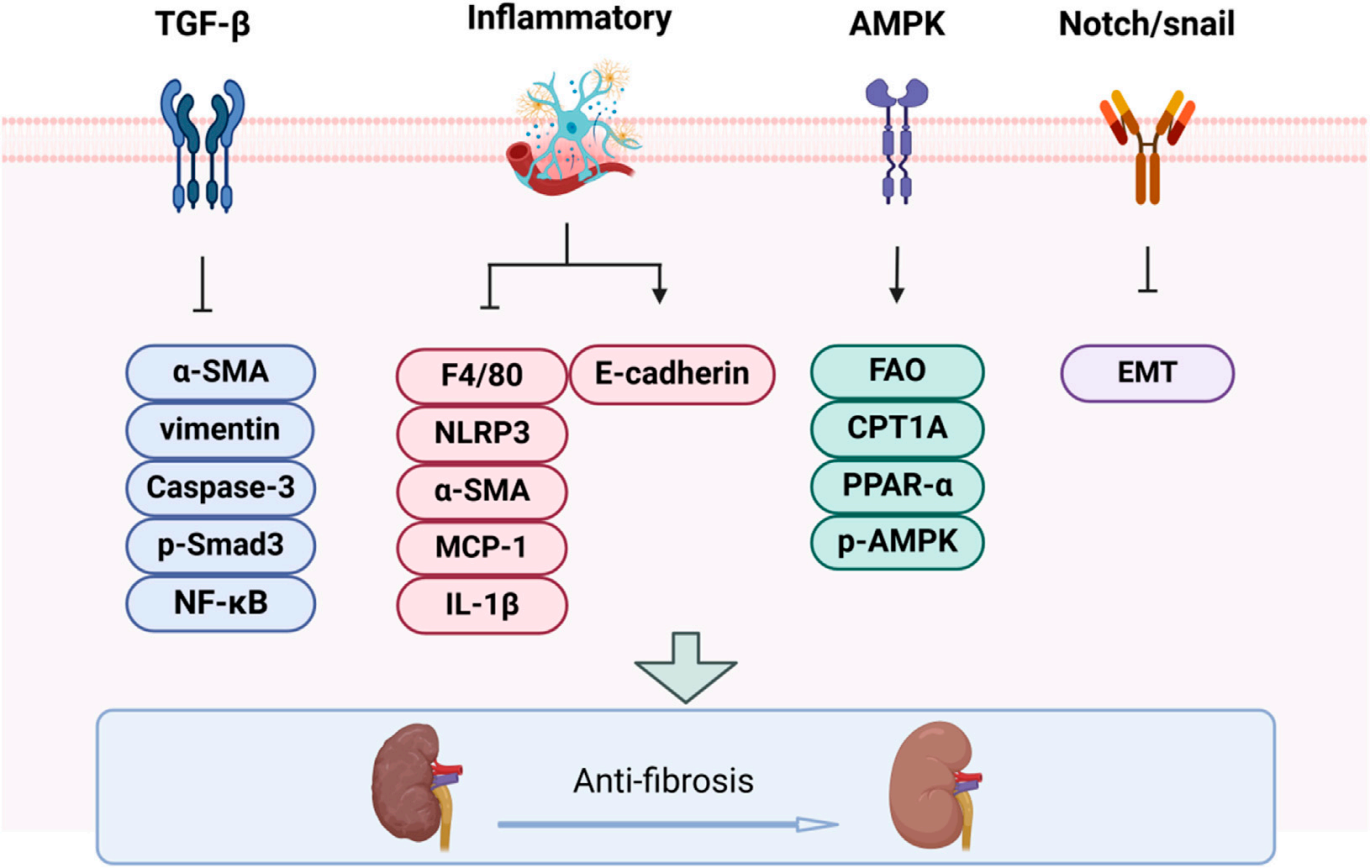
The impact of BBR on renal fibrosis progression.

Renal fibrosis (RF) is a prevalent feature of numerous chronic kidney diseases, characterized by enhanced extracellular matrix (ECM) synthesis and impaired degradation, which together facilitate intercellular matrix interactions. Therefore, BBR may confer renal protection by modulating these cellular interactions and their associated molecular pathways (Figure 2).

#### 3.2.1 TGF-β1/Smad signaling pathway

The TGF-β1/Smad signaling axis is pivotal in the progression of renal fibrosis and constitutes a core mechanism in its pathophysiology (Meng et al., 2016). In diabetic rat models, berberine exerts its anti-inflammatory and antifibrotic effects through the modulation of Smad7 expression. What’s more, it inhibits ECM deposition, alleviates glomerular basement membrane thickening, and mitigates renal tubular atrophy, thus attenuating various histopathological injuries associated with diabetes (Sun et al., 2015). In both diabetic rat and unilateral ureteral obstruction models, berberine treatment also attenuated the TGF-β/Smad3 signaling pathway, thereby mitigating renal fibrosis (Sun et al., 2015; Wang et al., 2014). Epiberberine, a potent derivative of berberine, ameliorates renal fibrosis by modulating the Agt-TGF-β/Smad2 signaling pathway (Xiao et al., 2021). Additionally, berberine mitigates tubulointerstitial fibrosis in DN through activation of the Nrf2 pathway and suppression of TGF-β/Smad/EMT signaling (Zhang et al., 2016).

#### 3.2.2 Inflammatory

The pathogenesis of RF primarily involves inflammation-driven injury to tubular epithelial cells (TECs), and modulation of the inflammatory response has the potential to attenuate or reverse the progression of RF (Song et al., 2024; Tan et al., 2023). BBR mitigated interstitial fibrosis and histopathological injury by suppressing NLRP3 inflammasome activation and IL-1β production. This intervention decreased the expression of MCP-1, F4/80, α-SMA, collagen I and collagen IV, while upregulating E-cadherin levels. Additionally, BBR alleviated serum creatinine and urea nitrogen abnormalities in both *in vivo* and *in vitro* models (Song et al., 2024; Tan et al., 2023).

#### 3.2.3 AMPK pathway

AMPK is instrumental in the initiation and progression of renal interstitial fibrosis. It regulates mitochondrial fatty acid oxidation (FAO) to compensate for diminished adenosine triphosphate (ATP) levels (Li et al., 2022). BBR treatment restored the phosphorylation of AMPK in the kidney and reversed the downregulation of FAO-associated proteins, including peroxiisome proliferator-activated receptor alpha (PPAR-α) and carnitine palmitoyltransferase 1A (CPT1A), both *in vitro* and *in vivo* (Tan et al., 2023). Other drugs may also exert their renal protective effects through AMPK-mediated mechanisms, such as autophagy (Dorweiler et al., 2007) and oxidative stress (Chertow et al., 2005).

#### 3.2.4 Notch/snail pathway

The Notch signaling pathway has been implicated in the regulation of cellular fibrosis, including EMT in DN, and is closely associated with TGF-β1 signaling (Yang et al., 2017). The expression of Snail1 is directly modulated by Notch signaling, and the Notch/Snail pathway plays a critical role in the pathogenesis of renal interstitial fibrosis in DN (Yang et al., 2017). BBR attenuated HG-induced EMT, downregulated Notch and Snail1 expression in renal tubular epithelial cells, and effectively inhibited renal interstitial fibrosis and tubular EMT both *in vitro* and *in vivo* (Yang et al., 2017).

### 3.3 Ischemia-reperfusion induced renal injury

IRI arises when blood supply is reinstated following a period of ischemia, leading to tissue and organ injury. In clinical settings, IRI represents a prevalent pathological mechanism (Jochmans et al., 2017). Renal ischemia is a primary etiology of acute renal failure (ARF), contributing to substantial morbidity and mortality. In addition, AKI has become a significant public health concern, with escalating incidence rates and limited therapeutic options (Neri et al., 2017). In both clinical and experimental studies, IRI precipitates multi-organ dysfunction, contributing to elevated mortality rates (Yu et al., 2013; Xie et al., 2017). Especially, a pioneering study by Yu et al. examined the renoprotective effects of BBR on human renal proximal tubular cells exposed to hypoxia/reoxygenation (H/R) injury. In this study, I/R induced significant damage to HK-2 cells, as demonstrated by reduced cell viability and heightened oxidative stress. Moreover, there was an upregulation of mitochondrial injury-related proteins, accompanied by an increase in both the apoptotic rate and expression of endoplasmic reticulum stress-associated proteins. BBR treatment was shown to significantly mitigate the abnormalities induced by H/R injury (Tait et al., 2015). In another study, Xie et al. demonstrated that berberine nanoparticles (BBR-NP) effectively protect TECs from renal IRI in rats. The research revealed that both BBR and BBR-NP alleviated renal damage, both functionally and morphologically, by reducing ROS, mitigating mitochondrial dysfunction, and attenuating apoptosis in renal cells (Yao et al., 2007).

### 3.4 Kidney aging

Although global life expectancy continues to rise, this does not necessarily correlate with an improvement in the overall quality of life. The aging process is marked by a gradual yet progressive decline in the functional capacity of multiple organs, impairing their ability to sustain baseline tissue homeostasis and adequately respond to physiological demands under stress (Chválová et al., 2007). Notably, El-Horany et al. explored the protective effects of BBR against kidney aging induced by D-galactose (D-gal) in rats.In this investigation, BBR significantly attenuated urea and serum creatinine (Scr) concentrations, reversed histopathological alterations in the kidneys, and restored redox balance, as evidenced by reduced levels of malondialdehyde (MDA) and 8-hydroxy-2′-deoxyguanosine, alongside the activation of heme oxygenase-1 (HO-1). Moreover, berberine markedly reduced serum levels of pro-inflammatory mediators, downregulated phosphatase and tensin homolog (PTEN) expression, while enhancing Akt hosphorylation and upregulating Bcl-2 protein levels (El-Horany et al., 2020).

### 3.5 Chemotherapy-induced renal injury

#### 3.5.1 Cisplatin-induced nephrotoxicity

Cisplatin (CP) is a prominent chemotherapeutic agent utilized in the treatment of solid tumors (Fuertes et al., 2003). As an alkylating compound, CP binds to DNA, inducing intrastrand crosslinking and the formation of adducts, which distort the DNA structure and hinder the process of DNA replication (Domitrović et al., 2013). Furthermore, cisplatin-induced cytotoxicity involves mitochondrial dysfunction, reduced ATPase activity, and disruption of cellular transport processes (Malaviya, 2016). In addition to its effects on tumor cells, cisplatin’s cytotoxic activity extends to normal somatic cells, particularly within the kidneys. The kidneys serve as the primary excretory route for cisplatin, with proximal tubular cells being the principal sites of drug accumulation (Yao et al., 2007). Interestingly, berberine has been shown to confer nephroprotection against cisplatin-induced renal injury, as evidenced by Domitrovi et al., through the reduction of oxidative and nitrosative stress, modulation of inflammatory responses, and suppression of apoptotic signaling (Vardi et al., 2013).

#### 3.5.2 Methotrexate-induced renal injury

Methotrexate (MTX), a potent anti-metabolite and folate antagonist, is administered at elevated doses for the treatment of various cancers, while lower doses are utilized for managing non-malignant conditions (Howard et al., 2016). MTX exerts its effects by inhibiting DNA synthesis through the suppression of dihydrofolate reductase (Hassanein et al., 2019). However, the nephrotoxic effects associated with MTX therapy represent a significant clinical challenge, primarily due to the direct tubular toxicity induced by its metabolite, 7-hydroxy-MTX. This nephrotoxicity can lead to compromised renal function, treatment delays, and substantial morbidity (Cheng et al., 2010). Hassanein et al. investigated the pivotal roles of the Bax/Bcl2/caspase-3, Keap1/Nrf2, and P38MAPK/NF-κB signaling pathways in mediating the protective effects of berberine against methotrexate-induced nephropathy (Cardoso et al., 2008).

#### 3.5.3 Doxorubicin-induced renal intoxication

Doxorubicin (DOX), a chemotherapeutic agent classified as an anthracycline, is a highly effective therapeutic used in the management of various hematological cancers and solid tumors (Tangpong et al., 2011). Despite its efficacy in cancer treatment, the clinical use of DOX is significantly constrained by its severe toxicities on off-target organs (Kalender et al., 2005; Bárdi et al., 2007). While the cardiac toxicity of DOX is well-documented, emerging research highlights its detrimental effects on additional organs, including the kidney, liver, and brain (Lopez-Novoa et al., 2011; Adil et al., 2016). Ibrahim Fouad G et al. found that DOX induced nephrotoxicity, characterized by marked elevations in serum urea, creatinine, and kidney injury molecule-1 (KIM-1) levels. Furthermore, DOX induced oxidative stress by elevating renal levels of MDA and hydrogen peroxide (H_2_O_2_), while concurrently reducing catalase (CAT) activity in the kidneys. DOX induced renal fibrosis and elevated levels of TGF-β1and increased collagen deposition. Additionally, DOX promoted apoptosis and inflammation in renal tissues, as demonstrated by upregulated expression of caspase-3 and NF-κB, respectively. These pathological alterations were significantly mitigated by concurrent BBR administration. Co-treatment with BBR effectively attenuated DOX-induced inflammatory response, oxidative stress, renal fibrosis and apoptosis (Ibrahim Fouad and Ahmed, 2021).

#### 3.5.4 Antibiotics-induced renal injury

Gentamicin (GM), an aminoglycoside antibiotic, is widely utilized in clinical settings for managing severe Gram-negative bacterial infections. Nonetheless, its clinical application is constrained by its potential to induce significant nephrotoxicity. Renal proximal tubule cells serve as the primary site of accumulation for aminoglycoside antibiotics, where they exert nephrotoxic effects via interaction with specific membrane transporters (Viljoen et al., 2019). Adil et al. explored the impact of BBR on gentamicin-induced nephrotoxicity in a rat model. In this study, GM administration resulted in a significant increase in BUN, Scr, and renal levels of MDA, KIM-1, nitric oxide (NO), NF-κB and neutrophil gelatinase-associated lipocalin (NGAL). Additionally, GM treatment markedly reduced renal superoxide dismutase (SOD) activity and depleted mitochondrial glutathione (GSH), nicotinamide adenine dinucleotide (NADH) dehydrogenase, Bcl-2, and cytochrome-c (Cyt-C) oxidase levels. BBR co-treatment reversed these alterations in a dose-dependent manner, restoring antioxidant defense mechanisms, attenuating inflammation, apoptosis, and markers of AKI, and modulating mitochondrial enzymatic functions (Jha et al., 2013).

### 3.6 Kidney stone

Kidney stone is a widespread health concern associated with a poor prognosis, often necessitating surgical intervention (Viljoen et al., 2019). Studies have demonstrated that BBR exerts its antiurolithic effects through multiple mechanisms. Similar to hydrochlorothiazide, BBR promotes diuresis, modulates urinary potential of hydrogen, and enhances Na⁺ and K⁺ excretion while reducing Ca^2^⁺ levels. In a rat model of ethylene glycol-induced calcium oxalate urolithiasis, BBR not only prevented and dissolved tubular crystal deposits but also significantly preserved renal function and mitigated oxidative stress. Additionally, *in vivo* experiments have confirmed that BBR increases urine output and alkalinity while lowering Ca^2^⁺ excretion, further supporting its nephroprotective potential (Bashir and Gilani, 2011).

### 3.7 Hypertensive renal impairment

In many developing countries, CKD is most typically associated with diabetes and/or hypertension (Jha et al., 2013). Wan et al. demonstrated in rats that BBR mitigates chronic renal damage caused by atherosclerotic renovascular disease through the suppression of the NF-κB signaling pathway. BBR significantly reduced blood pressure, low-density lipoprotein cholesterol, urinary albumin, and malondialdehyde levels. Additionally, treatment with BBR in the atherosclerotic renovascular disease model resulted in markedly lower expression levels of TGF-β1 compared to the untreated group, likely through the inhibition of NF-κB-DNA binding activity (Wan et al., 2013). Kishimoto et al. explored the impact of BBR on adipose tissue and renal function using 3T3-L1 cells and spontaneously hypertensive rats. *In vitro*, BBR supplementation suppressed the expression of CCAAT/enhancer-binding proteins α and β, as well as PPAR-γ, while downregulating PPAR target genes and inhibiting the differentiation of 3T3-L1 fibroblasts into adipocytes. In hypertensive rats, BBR administration led to a significant reduction in adipose tissue mass and alleviated renal injury (Kishimoto et al., 2015).

### 3.8 Uric acid nephropathy

Uric acid nephropathy, or gouty nephropathy, is a metabolic disorder primarily driven by hyperuricemia. Epidemiological data indicate a rising prevalence of Uric acid nephropathy, closely mirroring the increasing incidence of hyperuricemia, a trend associated with improvements in lifestyle and overall quality of life (Gong et al., 2024). The study demonstrated that BBR exerts an anti-hyperuricemic effect, partially through the modulation of urate transporter expression and the inhibition of the JAK2/STAT3 signaling pathway (Pan et al., 2023). Gong et al. Explored BBR binds to red blood cells, is identified and internalized by monocytes, and subsequently accumulates in damaged renal tissue. Upon entry, it activates AMPK, suppresses NF-κB-mediated inflammatory signaling, inhibits macrophage polarization (Imenshahidi and Hosseinzadeh, 2016).

Recent studies have found that BBR can also relieve renal fibrosis through the gut–kidney axis. Pan et al. identified gut microbiota modulation as a key nephroprotective mechanism of berberine. Their study explored BBR’s therapeutic effects in CKD and demonstrated that it reduces gut-derived uremic toxins while reshaping intestinal microbiota composition. Notably, BBR was found to inhibit the microbial metabolism of tyrosine, thereby suppressing p-cresol production, a process potentially linked to its ability to restrain *Clostridium* proliferation. These findings provide the first evidence that BBR mitigates CKD through the gut-kidney axis, highlighting its considerable potential as a therapeutic agent for CKD management (Liu et al., 2016).

## 4 Toxicity of BBR

BBR is generally regarded as safe at standard dosages, exhibiting minimal toxicity and adverse effects (Imenshahidi and Hosseinzadeh, 2019; Chitra et al., 2013). Clinical studies have primarily reported mild gastrointestinal disturbances, such as diarrhea and constipation, as the most common side effects (Germoush and Mahmoud, 2014). On the one hand, BBR has the potential to mitigate the adverse effects and toxicities associated with various chemotherapeutic and analgesic agents, including cyclophosphamide and cisplatin (Vardi et al., 2013; Feng et al., 2018; Holy et al., 2009). On the other hand, under certain conditions, BBR may induce adverse effects. For instance, a dose of 10 mg/kg BBR has been shown to impair immune function in murine models[98–100]. Additionally, interactions between BBR and macrolides or statins can result in cardiac arrhythmias and diminish the therapeutic efficacy of these drugs (Zhi et al., 2015). To date, there have been no documented instances of severe adverse reactions associated with oral BBR administration in clinical settings. In addition, BBR has been deemed safe for the majority of human subjects in both short-term and long-term clinical trials. In conclusion, based on conventional dosages and indications, BBR is considered safe for oral administration.

## 5 Conclusion

Renal and urological disorders can affect individuals of any age and at any time. In this review, we aim to provide a comprehensive analysis of BBR’s protective effects across a range of renal pathologies. The renoprotective effects of berberine are largely attributed to its ability to mitigate oxidative stress, suppress inflammatory pathways, and inhibit apoptotic processes, as evidenced by the reviewed literature (Table 1). Several studies have demonstrated that BBR exerts substantial renoprotective effects across a range of renal disorders, including diabetic nephropathy, renal fibrosis, renal aging, chemotherapy-induced renal injuryand kidney toxicity induced by chemotherapy and antibiotics. These findings suggest that BBR holds significant and evolving therapeutic potential. Furthermore, extensive research on BBR’s clinical use in humans has established its safety profile, even at higher dosages, in the treatment of various diseases. Consequently, further investigations into the renoprotective properties of berberine in patients with AKI and CKD are warranted. Therefore, future research should prioritize additional clinical trials as well as *in vivo* and *in vitro* studies. It is anticipated that the accumulation of robust scientific evidence will significantly enhance the guidance for clinical treatment and prevention. Of course BBR’s potential use in combination therapies with other nephroprotective agents or its role in chronic kidney disease management should also be strengthened. Concurrently, these advancements will strengthen the theoretical foundation for the management of AKI and CKD, ultimately facilitating the standardization of berberine-based therapeutic strategies for various renal disorders.

**TABLE 1:** An overview of the renoprotective effects of berberine across various renal diseases and the underlying molecular mechanisms.

Disease	Animals or cells model	Effects	Pathway	References
Diabetic nephropathy	STZ-induced DN rats	Mitigate oxidative stress, suppress inflammatory pathways and inhibit apoptotic processes	TLR4/NF-κB pathway	Zhu et al. (2018)
Diabetic nephropathy	STZ-induced DN rats	Suppress the elevation of fibronectin levels triggered by the S1P2 receptor	SphK1/S1P/S1P2 pathway	Huang et al. (2012)
Diabetic nephropathy	STZ-induced DN rats	Inhibit renal inflammation blocking the upregulation of pro-inflammatory cytokines (IL-1β, TNFα) and chemokine (MCP-1)	TGF-β/Smad3 pathway	Sun et al. (2015)
Diabetic nephropathy	High-fat diet and STZ induced DN rats and Leprdb/db mice	Inhibit glomerular mesangial matrix expansion, activate autophagy and activate of AMPK phosphorylation	Activate AMPK phosphorylation	Zhang et al. (2020)
Diabetic nephropathy	High-sugar, high-fat diet and STZ induced DN rats	Regulate NLRP3-Caspase-1-GSDMD signalling pathway, inhibit kidney cell pyroptosis and antagonize DN inflammatory damage	Nrf2-NLRP3-Caspase-1-GSDMD pathway	Ding et al. (2021)
Diabetic nephropathy	High-fat diet and STZ induced DN rats and HK_2_ cell	Suppress the NLRP3 inflammasome	NLRP3 inflammasome	Ma et al. (2022)
Diabetic nephropathy	DN model KKAy mice and Mouse renal tubular epithelial cells (mRTECs)	Suppress renal tubular epithelial EMT, reduce renal interstitial fibrosis	Notch/snail pathway	Yang et al. (2017)
Renal fibrosis	UUO in rats	Inhibit of oxidative stress, inflammatory responses, and TGF-β1/pSmad3 signalling	TGF-β1/pSmad3 pathway	Wang et al. (2014)
Renal fibrosis	db/db mice and high-glucose induced glomerular mesangial cells (GMCs)	Reduce Agt, TGFβ1, and Smad2 expression *in vitro* and *in vivo*	Agt-TGFβ/Smad2 pathway	Xiao et al. (2021)
Renal fibrosis	STZ-induce diabetic mice and normal rat kidney tubular epithelial (NRK 52E) cells	Activate Nrf2 pathway and inhibit TGF-β/Smad/EMT signaling activity	TGF-β/Smad/EMT pathway	Zhang et al. (2016)
Renal fibrosis	UUO in rats and HK-2 cells	Deactivate of the NLRP3 inflammasome and protection of TECs by reversing defective FAO	Activate AMPK	Tan et al. (2023)
Renal fibrosis	High-fat diet (HFD)-fed mice	Suppress macrophage migration towards adipocytes by activating SIRT3	Activate the deacetylase SIRT3	Li et al. (2022)
IRI induced injury	HK-2 cells	Attenuat oxidative stress and apoptosis link to mitochondrial stress	ER stress pathways	Yu et al. (2013)
IRI induced injury	IRI in rats	Suppress ROS, mitochondrial dysfunction and apoptosis	ROS and apoptosis	Xie et al. (2017)
Kidney aging	D-gal-induced kidney aging in rats	Reduce the serum levels of pro-inflammatory mediators, along with downregulation of PTEN expression, enhanced Akt activity	PTEN/Akt pathway	El-Horany et al. (2020)
Chemotherapy induced renal injury	CDDP-induced renal injury in BALB/cN mice	Suppress oxidative, inflammation and apoptosis	Oxidative, inflammation, apoptosis	Domitrović et al. (2013)
Chemotherapy induced renal injury	MTX-induced renal injury in rats	Anti-oxidant and anti-inflammatory and anti-apoptotic	Bax/Bcl2/caspase-3, Keap1/Nrf2, and P38MAPK/NF-κB pathway	Hassanein et al. (2019)
Chemotherapy induced renal injury	DOX-induced acute toxicity in rats	Suppress oxidative stress	Oxidative stress	Ibrahim Fouad and Ahmed (2021)
Antibiotics-induced renal injury	GNT-induced renal intoxication in rats	Attenuat of oxidative stress, inflammation, apoptosis and mitochondrial dysfunction	Oxidative, inflammation, apoptosis	Adil et al. (2016)
kidney stone	Ca-oxalate urolithiasis induced by ethylene glycol in rats	Attenuat renal oxidative stress	Oxidative stress	Bashir and Gilani (2011)
Hypertensive renal impairment	Renal injury induced by atherosclerotic renovascular disease in rats	Inhibit the NF-κB signalling pathway, attenuat renal oxidative stress	NF-κB signalling pathway, oxidative stress	Borghi et al. (2020)
Hypertensive renal impairment	3T3-L1 cells and spontaneously hypertensive rats	Suppress of PPAR target genes and inhibit of differentiation of 3T3-Ll fibroblast to adipocytes	PPAR signalling pathway	Lin et al. (2021)
Uric acid nephropathy	PO/HX induced Uric acid nephropathy in mice	Regulate urate transporter expressions and JAK2/STAT3 signaling pathway	JAK2/STAT3 signaling pathway	Pan et al. (2023)
Uric acid nephropathy	THP-1 cells and PO/HX induced Uric acid nephropathy in mice	Activate AMPK, inhibit the activation of inflammatory pathway NF-κB and regulate macrophage polarization	NF-κB signalling pathway	Imenshahidi and Hosseinzadeh (2016)

## Glossary

BBR: Berberine
AKI: Acute kidney injury
CKD: Chronic kidney disease
NSAIDs: Nonsteroidal anti-inflammatory drugs
I/R: Ischemia-reperfusion
ROS: Reactive oxygen species
IRI: Ischemia-reperfusion injury
UUO: Unilateral ureteral obstruction
TGF-β: Transforming growth factor-β
IL-1β: Interleukin-1β
IL-6: Interleukin-6
SASP: Senescence-associated secretory phenotype
NF-κB: Nuclear factor-κB
NLRP3: NOD-like receptor thermal protein domain associated protein 3
Ang II: Angiotensin II
STZ: Streptozotocin
TLR4: Toll-like receptor 4
LC3: Light chain 3
Bcl-XL: B-cell lymphoma-XL
Atg5: Autophagy-related protein
Atg7: Autophagy-related protein 7
DN: Diabetic nephropathy
ESRD: End-stage renal disease
Cr: Creatinine
BUN: Blood urea nitrogen
HG: High glucose
FN: Fibronectin
S1P: Sphingosine 1-phosphate
AMPK: Adenosine 5′-monophosphate (AMP)-activated protein kinase
GSDMD: Gasdermin D
Nrf2: Nuclear factor erythroid 2-Related Factor 2
EMT: Epithelial-to-mesenchymal transition
RF: Renal fibrosis
ECM: Extracellular matrix
TECs: Tubular epithelial cells
FAO: Fatty acid oxidation
ATP: Adenosine triphosphate
PPAR-α: Peroxiisome proliferator-activated receptor alpha
CPT1A: Carnitine palmitoyltransferase 1A
ARF: Acute renal failure
H/R: Hypoxia/reoxygenation
HK-2: Human kidney-2
BBR-NP: Berberine nanoparticles
D-gal: D-galactose
MDA: Malondialdehyde
HO-1: Heme oxygenase-1
PTEN: Phosphatase and tensin homolog
Bcl-2: B-cell lymphoma-2
CP: Cisplatin
MTX: Methotrexate
DOX: Doxorubicin
KIM-1: Kidney injury molecule-1
H2O2: Hydrogen peroxide
CAT: Catalase
GM: Gentamicin
NO: Nitric oxide
NGAL: Neutrophil gelatinase-associated lipocalin
SOD: Superoxide dismutase
GSH: Glutathione
NADH: Nicotinamide adenine dinucleotide
Cyt-C: Cytochrome-c
